# Faunal response to sea‐level and climate change in a short‐lived seaway: Jurassic of the Western Interior, USA

**DOI:** 10.1111/pala.12278

**Published:** 2017-02-08

**Authors:** Silvia Danise, Steven M. Holland

**Affiliations:** ^1^ Department of Geology University of Georgia 210 Field Street Athens GA 30602‐2501 USA; ^2^ School of Geography, Earth & Environmental Sciences Plymouth University Drake Circus Plymouth PL4 8AA UK

**Keywords:** Jurassic, climate change, sea level, cooling event, benthos, stratigraphic palaeobiology

## Abstract

Understanding how regional ecosystems respond to sea‐level and environmental perturbations is a main challenge in palaeoecology. Here we use quantitative abundance estimates, integrated within a sequence stratigraphic and environmental framework, to reconstruct benthic community changes through the 13 myr history of the Jurassic Sundance Seaway in the western United States. Sundance Seaway communities are notable for their low richness and high dominance relative to most areas globally in the Jurassic, and this probably reflects steep temperature and salinity gradients along the 2000 km length of the Seaway that hindered colonization of species from the open ocean. Ordination of samples shows a main turnover event at the Middle–Upper Jurassic transition, which coincided with a shift from carbonate to siliciclastic depositional systems in the Seaway, probably initiated by northward drift from subtropical latitudes to more humid temperate latitudes, and possibly global cooling. Turnover was not uniform across the onshore–offshore gradient, but was higher in offshore environments. The higher resilience of onshore communities to third‐order sea‐level fluctuations and to the change from a carbonate to a siliciclastic system was driven by a few abundant eurytopic species that persisted from the opening to the closing of the Seaway. Lower stability in offshore facies was instead controlled by the presence of more volatile stenotopic species. Such increased onshore stability in community composition contrasts with the well‐documented onshore increase in taxonomic turnover rates, and this study underscores how ecological analyses of relative abundance may contrast with taxonomically based analyses. We also demonstrate the importance of a stratigraphic palaeobiological approach to reconstructing the links between environmental and faunal gradients, and how their evolution through time produces local stratigraphic changes in community composition.

The deep‐time fossil record can be used to understand the ecological and evolutionary responses of species to changes in their environment, and provides an important tool for identifying those factors that might impart resilience in the face of environmental change (Willis *et al*. 2010). Studies of long‐term change in regional communities have shown that turnover of ecosystems varies markedly, ranging from long‐lived relative faunal stability to brief elevated turnover (Brett & Baird 1995; Behrensmeyer *et al*. 1997; Patzkowsky & Holland 1997; DiMichele *et al*. 2004; Holland & Patzkowsky 2007; Ivany *et al*. 2009; Kowalewski *et al*. 2015). Stability can result from strong ecological interactions (Mougi & Kondoh 2012), broad geographical range (Payne & Finnegan 2007), wide niche breadth (Jackson 1974), high population abundance (McKinney *et al*. 1996) and dispersal sufficient to allow habitat tracking (Brett *et al*. 2007; Zuschin *et al*. 2014). Understanding the link between biotic turnover and environmental change remains a challenge in palaeoecology, particularly because much environmental change has a minimal effect on turnover (e.g. Morris *et al*. 1995), whereas some environmental change appears to trigger marked turnover, suggesting possible threshold effects (e.g. Hesselbo *et al*. 2007; Zhang *et al*. 2009; Finnegan *et al*. 2012; Danise *et al*. 2013, 2015). Variations in community composition along onshore–offshore water‐depth gradients (Holland & Patzkowsky 2004; Scarponi & Kowalewski 2004) raises the possibility of observing differential responses to communities to the same environmental perturbation (Holland & Patzkowsky 2007; Bonelli & Patzkowsky 2008). Distinguishing between true temporal changes in community composition and stratigraphical variation in community composition resulting from local changes in depositional environment requires a stratigraphic palaeobiological approach of controlled sampling within depositional environments of successive depositional sequences (Holland 1995, 2000; Patzkowsky & Holland 2012). This approach of interpreting the fossil record against a sequence stratigraphical framework allows a deeper analysis of factors underlying the change of fossil communities through time and space (e.g. Scarponi & Kowalewski 2004; Dominici & Kowalke 2007; Tomašových *et al*. 2014).

Here we present a species‐level study of marine benthic community response to sea‐level and climate change from the Middle–Upper Jurassic Sundance Seaway of the western United States. Globally, the Jurassic was characterized by rapidly increasing ecospace utilization and biological diversification, and by the origin and radiation of the major groups that constitute modern marine ecosystems (Vermeij 1977; Sepkoski 1981; Bush & Bambach 2011; Finnegan *et al*. 2011). Despite the interest in Jurassic ecosystems, only one previous study has examined turnover patterns in Jurassic regional communities, and it reported near‐stasis for approximately 20 myr (Tang & Bottjer 1996). Developed within marine deposits of the Jurassic Sundance Seaway are eight third‐order, unconformity‐bounded, depositional sequences, collectively representing approximately 13 myr (Pipiringos 1968; Pipiringos & O'Sullivan 1978; Brenner & Peterson 1994; McMullen *et al*. 2014; Clement & Holland 2016). Superimposed on these cyclical changes in sea level is a transition from subtropical arid climates into progressively more humid conditions (Boucot *et al*. 2013), resulting in part from the northward migration of the North American plate (Johnson 1992). These environmental and climatic changes make the Sundance Seaway an ideal site to investigate the linkage between environmental change and turnover.

## Geological Setting

### Palaeogeography and palaeoclimate

**Figure 1:**
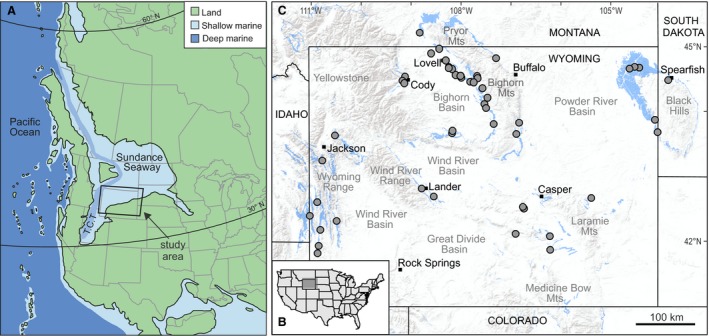
Palaeogeography and location map of the study area. A, palaeogeographical reconstruction of western North America in the Middle Jurassic (Bajocian Stage ~170 Ma), with location of study area. Map based on maps produced by R. Blakey (http://deeptimemaps.com/western-interior-seaway-map-list/; accessed 6 January 2015). *Abbreviation*: T.C.T: Twin Creek Trough. B, map of the United States with study area indicated in dark grey. C, map of Wyoming and surrounding states, with sampled localities indicated by grey dots. Colour online.

**Figure 2:**
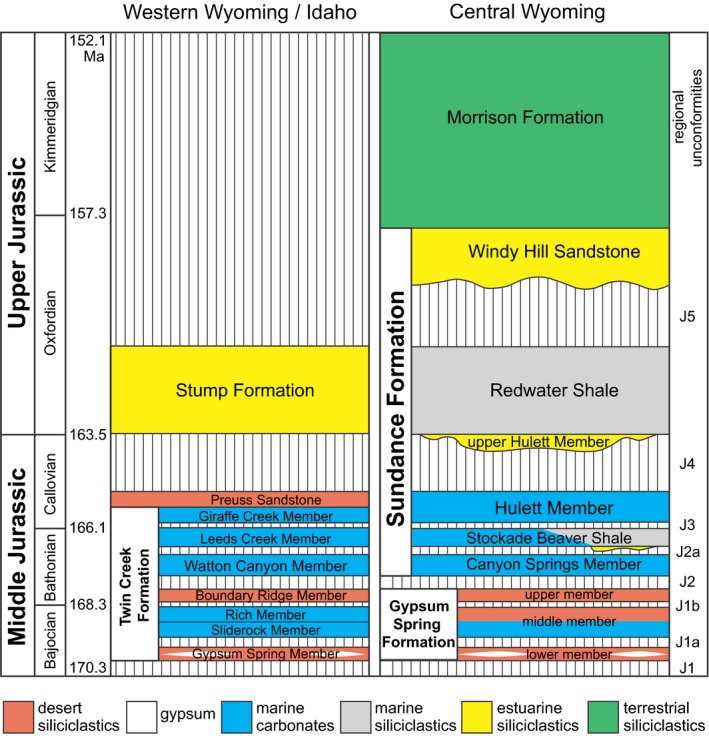
Chronostratigraphical and sequence stratigraphical framework of the Jurassic Twin Creek Formation in western Wyoming and Idaho and the Sundance Formation in central and eastern Wyoming. Chronostratigraphy of units is based on Pipiringos & O'Sullivan (1978), Imlay (1952, 1967, 1980), Brenner & Peterson (1994), Kvale *et al*. (2001) and Parcell & Williams (2005). Shown at right are unconformities recognized by Pipiringos (1968) and Pipiringos & O'Sullivan (1978), as recently modified by McMullen *et al*. (2014) and Clement & Holland (2016). Absolute time scale is from Cohen *et al*. (2013). Colour online.

The study interval spans the Middle to Upper Jurassic (Bajocian to Oxfordian; ~170–155 Ma) marine and continental deposits from siliciclastic, carbonate, and evaporite systems in the Sundance Seaway of Wyoming and adjacent states (Fig. 1). The Sundance Seaway developed in an elongated, retro‐arc foreland basin connected to the Pacific Ocean (Fig. 1), with the Cordilleran volcanic arc to the west and the North American craton to the east (Peterson 1954; Brenner & Peterson 1994; Lawton 1994; Bjerrum & Dorsey 1995). The axis of the basin extended from southern Utah northward into northern British Columbia, a length of nearly 2000 km. Thrust faulting to the west during the Middle Jurassic created a foredeep in Utah, eastern Idaho and western Wyoming, within which the Twin Creek Formation was deposited (Fig. 2). To the east of this foredeep, a west to north‐westward‐facing ramp developed, upon which the Gypsum Spring and Sundance formations were deposited (Fig. 2).

Palaeogeographical reconstructions place Wyoming from 22–30° N (Kocurek & Dott 1983; Saleeby & Busby‐Spera 1992) to 35–40° N in the Middle to Upper Jurassic (Blakey 2014). Northward drift of North America during the Jurassic (May & Butler 1986) caused Wyoming and surrounding areas to move northward from the subtropical arid belt into progressively more humid climates, characterized by winter‐wet conditions (Johnson 1992; Rees *et al*. 2000; Boucot *et al*. 2013). This northward drift also moved the region from the belt of the easterly trade winds into the mid‐latitudes with their westerly winds (Kocurek & Dott 1983).

### Stratigraphy and depositional environments

Most studies of the Sundance Seaway predate modern sequence stratigraphical concepts (e.g. Imlay 1947, 1956, 1967; Peterson 1954). Three recent studies on the Gypsum Spring and Sundance formations of the Bighorn Basin, Wyoming, have developed the sequence stratigraphical framework used in this study (Parcell & Williams 2005; McMullen *et al*. 2014; Clement & Holland 2016). We have extended this across Wyoming, into adjacent parts of South Dakota, Montana and Idaho; this extended sequence stratigraphical framework will be published separately.

The Gypsum Spring and Sundance formations are exposed in Wyoming along the flanks of Late Cretaceous to Cenozoic uplifts, including the Black Hills, Pryor Mountains, Bighorn Mountains, Wind River Mountains, Wyoming Range and Laramie Mountains. The Gypsum Spring Formation, with a maximum thickness of about 80 m, was deposited on a north‐westward‐dipping mixed evaporate–carbonate–siliciclastic ramp, with depositional environments that include distal ooid shoals, open shallow subtidal, restricted shallow subtidal, peritidal, salinas and desert mud flat to sabkha (Clement & Holland 2016). It is divided informally into lower, middle and upper members (Parcell & Williams 2005; Clement & Holland 2016), and only the middle member is fossiliferous.

The Sundance Formation is approximately 100 m thick, and records cyclical deposition on a north‐westward‐dipping mixed siliciclastic–carbonate system. The complex alternation of mudstones, limestones and sandstones has led to a complex lithostratigraphical nomenclature (Imlay 1947, 1980; Pipiringos 1968; Wright 1973; Kvale *et al*. 2001). We apply the framework developed by McMullen *et al*. (2014) for the Bighorn Basin to the entire area of study (Fig. 2), as well as their facies model. McMullen *et al*. (2014) divided the Sundance Formation into six members. The Canyon Springs and lower Hulett members are each dominated by carbonate rocks, deposited mainly in the shallow subtidal and on ooid shoals (McMullen *et al*. 2014). The Stockade Beaver Shale was deposited on a mixed carbonate–siliciclastic shelf, with offshore, carbonate mudstone facies to the west, and siliciclastic offshore and offshore transition facies to the east. The upper Hulett Member is a siliciclastic incised‐valley fill capped by transgressive ooid shoal facies. The Redwater Shale Member was deposited on a wave‐dominated siliciclastic shelf, and the Windy Hill Sandstone was deposited in a tidal estuary. The Windy Hill grades upward through progressive loss of tidal influence into overlying coastal plain deposits of the Upper Jurassic Morrison Formation (lower Oxfordian to lower Tithonian: Imlay 1962, 1980; Pipiringos 1968; Kowallis *et al*. 1998). All members of the Sundance Formation are fossiliferous.

The Twin Creek Formation is exposed in the Wyoming Range of westernmost Wyoming and eastern Idaho, and comprises a thick sequence of marine carbonates and shales deposited on westward‐dipping mixed evaporate–carbonate ramp. The Twin Creek thickens to the west, where it reaches a maximum thickness of about 700 m in the Twin Creek Trough. The facies models of McMullen *et al*. (2014) and Clement & Holland (2016) can be readily applied to the Twin Creek Formation, with the addition of carbonate offshore and deep subtidal facies not present to the east. The Twin Creek Formation is subdivided into seven members that were deposited in spectrum of environments ranging from desert mudflat and sabkha to carbonate offshore (Fig. 2; Imlay 1967). The Twin Creek Formation is overlain by middle Callovian to Oxfordian Preuss and Stump formations, deposited in hypersaline intertidal mud flats (Kocurek & Dott 1983) and deltas (Patterson‐Wittstrom 1980), and they are in turn unconformably overlain by the Cretaceous Gannett Group (Rubey 1973; Patterson‐Wittstrom 1980).

The marine Jurassic of the Sundance Seaway comprises eight main unconformity‐bounded depositional sequences (Fig. 2). Each is named for its underlying sequence boundary, which corresponds in most cases to a previously recognized regional unconformity defined by a chert‐pebble horizon (Fig. 2; Pipiringos 1968; Pipiringos & O'Sullivan 1978; Parcell & Williams 2005; McMullen *et al*. 2014; Clement & Holland 2016). We have correlated these unconformity‐bounded depositional sequences across Wyoming and into the Twin Creek Formation, resulting in a regional sequence stratigraphical framework.

The stage assignments from all the studied units derive from previous biostratigraphical studies, particularly those of Imlay (1947, 1956, 1967, 1980). The biostratigraphical zonation and correlation of the Gypsum Spring Formation is reviewed in Clement & Holland (2016), that of the Sundance is summarized by Calloman (1982) and Kvale *et al*. (2001), and Imlay (1967) documents the biostratigraphy of the Twin Creek Formation.

## Method

### Censuses and stratigraphical context

Faunal censuses were obtained from marine rocks of the Gypsum Spring, Sundance and Twin Creek formations at 44 localities in Wyoming, Montana and South Dakota during 2014–2016 (Fig. 1). Censuses were conducted on bedding surfaces or vertical surfaces in the field, and by collecting samples of approximately 7.5 l, which were counted in the laboratory. In all cases, representative samples were collected to establish species‐level identifications.

Fossils were identified to species wherever possible, although only genus‐ and family‐level identifications were possible in some cases. The minimum number of individuals was calculated following standard approaches (see Patzkowsky & Holland 2012), except for echinoids and crinoids. As these commonly disarticulate into numerous ossicles, the number of echinoderm individuals was estimated by dividing the number of ossicles and spines by 100. The total dataset contains 184 samples, 120 taxa and 11347 individuals, and includes 82 species of bivalve, 15 gastropod species, 9 echinoderm species (crinoids and echinoids), 7 serpulid species, 2 brachiopod species, 2 bryozoan species, 1 belemnite species, 1 coral species and 1 decapod species (Danise & Holland 2017). Unusually for the Jurassic, ammonites are rarely encountered in the study area and were not present in any of our samples. The data set is dominated by bivalves, which represent 88.6% of all individuals.

Taxonomic identifications were based on Meek & Hayden (1865), White (1880, pls 37–38), Newton *et al*. (1880), Stanton (1899), Whitfield & Hovey (1906), Clark & Twitchell (1915), Koch (1962), Imlay (1964, 1967), Sohl (1965), Hess (1972) and Tang *et al*. (2000). Identifications were also accomplished by comparison with type specimens of bivalves and gastropods from the Sundance and Twin Creek formations at the Smithsonian Institution's National Museum of Natural History.

**Figure 3:**
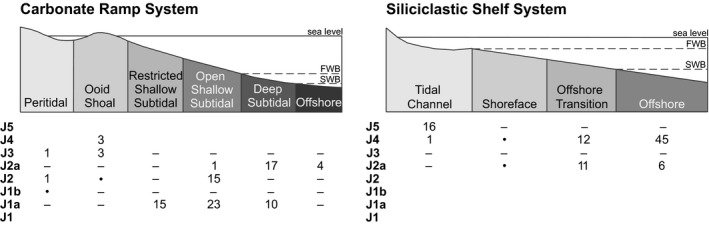
Time–environment plot showing number of samples grouped by depositional sequence and environment. Combinations lacking samples generally reflect those sequences containing either the siliciclastic system or the carbonate system, as well as the unavailability of particular depositional environments in certain sequences (e.g. shoreface environments are absent in some sequences and are unfossiliferous where they do occur). The J4 ooid shoal is an ephemeral transgressive carbonate environment within a siliciclastic system. *Symbols*: – depositional environment not present; • depositional environment unfossiliferous; blank, depositional system not present.

At each locality, stratigraphical columns were logged for lithology, bedding, sedimentary structures, and trace fossils. These were interpreted in terms of depositional environment, and stratigraphical intervals were assigned to specific sedimentary facies and depositional sequences (Fig. 3). Two main depositional systems are distinguished, a siliciclastic shelf and a carbonate ramp. The siliciclastic system contains facies representing four progressively landward environments: offshore (deeper than storm wave base), offshore transition, shoreface (shallower than fair‐weather wave base) and tidal channel (from a tidal estuary system). The carbonate ramp system comprises facies from six depositional environments: offshore (below storm wave base), deep subtidal (between storm wave base and fair‐weather wave base), open shallow subtidal (shallower than fair‐weather wave base), restricted shallow subtidal, ooid shoal and peritidal. Of the eight total depositional sequences, only six (J1a, J2, J2a, J3, J4, J5) are fossiliferous. Some sedimentary environments were typically unfossiliferous (e.g. shoreface) or poorly fossiliferous (e.g. restricted shallow subtidal, peritidal), each sequence tends to preserve either the siliciclastic system or the carbonate system but not both, and not all facies within a given system and sequence are exposed within the study area. As a result, it is not possible to sample the time‐environment matrix completely, as is often the case in the fossil record (Smith *et al*. 2001; Patzkowsky & Holland 2012).

### Analytical methods

Before numerical analysis, all taxa occurring in only one sample and all samples containing only one taxon were removed to prevent the distortion they create in multivariate analyses. The resulting culled dataset contains 157 samples, 70 taxa and 8466 individuals. Of the species, 49 are bivalve, with 8 gastropods, 6 serpulids, 5 crinoids, 1 belemnite and 1 brachiopod species. The culled data set is also dominated by bivalves, which represent 85.3% of all individuals. The median sample size is 40 individuals (minimum of 11, maximum of 306). Prior to multivariate analysis, a percent transformation within samples was performed, followed by a log transformation to lessen the dominating influence of the most abundant taxa.

Data were ordinated using nonmetric multidimensional scaling (NMDS), a useful ordination method for detecting patterns of co‐occurrence among taxa as well as ecological gradients (Legendre & Legendre 1998). Ordinations used Bray–Curtis dissimilarity, three axes, 100 restarts to prevent reaching a local optimum, and weighted averaging to calculate taxon scores. NMDS ordination was performed with the metaMDS() function in the vegan package of R (R Core Team 2014). This function rotates the NMDS solution via principal components analysis such that NMDS axis 1 reflects the principal source of variation, and so on, as is characteristic of eigenvalue methods. Detrended correspondence analysis was also performed, but NMDS results better segregated samples of clearly different composition, as is sometimes the case (Patzkowsky & Holland 2012; Tyler & Kowalewski 2014). NMDS ordinations were also performed separately for Middle Jurassic (J1a–J3) and Upper Jurassic (J4–J5) samples, following the same protocols as in the ordination of the total data set. Stress values were 0.08 for the NMDS ordination of the entire dataset, and respectively, 0.08 and 0.09 for the Middle and Upper Jurassic ordinations, values that suggest little distortion (Clarke & Warwick 2001).

A two‐way cluster analysis was performed to describe groups of samples with similar faunal compositions (Q‐mode) and groups of taxa that tend to co‐occur (R‐mode). The clustering algorithm used agglomerate nesting, coupled with Ward's method, which adds samples to existing clusters that minimize the total sum of squares. Ward's method tends to produce dendrograms with well‐defined clusters (Legendre & Legendre 1998). Biofacies were defined using Q‐mode cluster analysis (cf. Ludvigsen *et al*. 1986). The two‐way cluster analysis was performed using the hclust() function in R's vegan package and the heatmap() function of the latticeExtra R package.

For each depositional environment in each sequence, and for each biofacies, species richness (S) and the Simpson index (1‐D) were calculated from the unculled dataset. The Simpson index, calculated as 1−Ʃ(*p*_*i*_)^2^, where *p* is the proportional abundance of species *i*, is an unbiased measure of evenness which ranges from zero (one taxon dominates the community completely) to one (all taxa have equal abundance; Lande 1996). The t.test() function in R was used to generate 95% confidence intervals on mean evenness. To allow comparison with previous studies, biofacies richness was also measured by: (1) standardizing samples with rarefaction to 30 individuals, *S*_rar30_ (Krebs 1999); (2) combining all samples in each biofacies, *S*_pool_; (3) standardizing with Shareholder Quorum Subsampling (SQS) to a quorum of 0.5, *S*_SQS0.5_ (Alroy 2010). Sample rarefaction and 95% confidence intervals were performed with the program Analytic Rarefaction (Hunt Mountain Software 2012)

Similarity measures were used to quantify temporal turnover in taxonomic composition (*see* Anderson *et al*. 2011). Pairwise comparisons of groups of samples from the same depositional environment (e.g. shallow subtidal) but different depositional sequences (e.g. J1a vs J2) were used to produce a distance matrix. Jaccard dissimilarity (Jaccard 1912) was used for presence–absence data, and Bray–Curtis dissimilarity (Bray & Curtis 1957) was used for relative‐abundance data. Similarities were calculated using vegdist() function of the vegan package in R, but were inverted such to express similarity where a value of 1.0 indicates identical groups and 0.0 represents completely different groups. The t.test() function in R was used to generate 95% confidence intervals on similarity. Similarity was calculated only for environments containing more than two samples per sequence.

## Results

### NMDS ordination of the entire dataset

**Figure 4:**
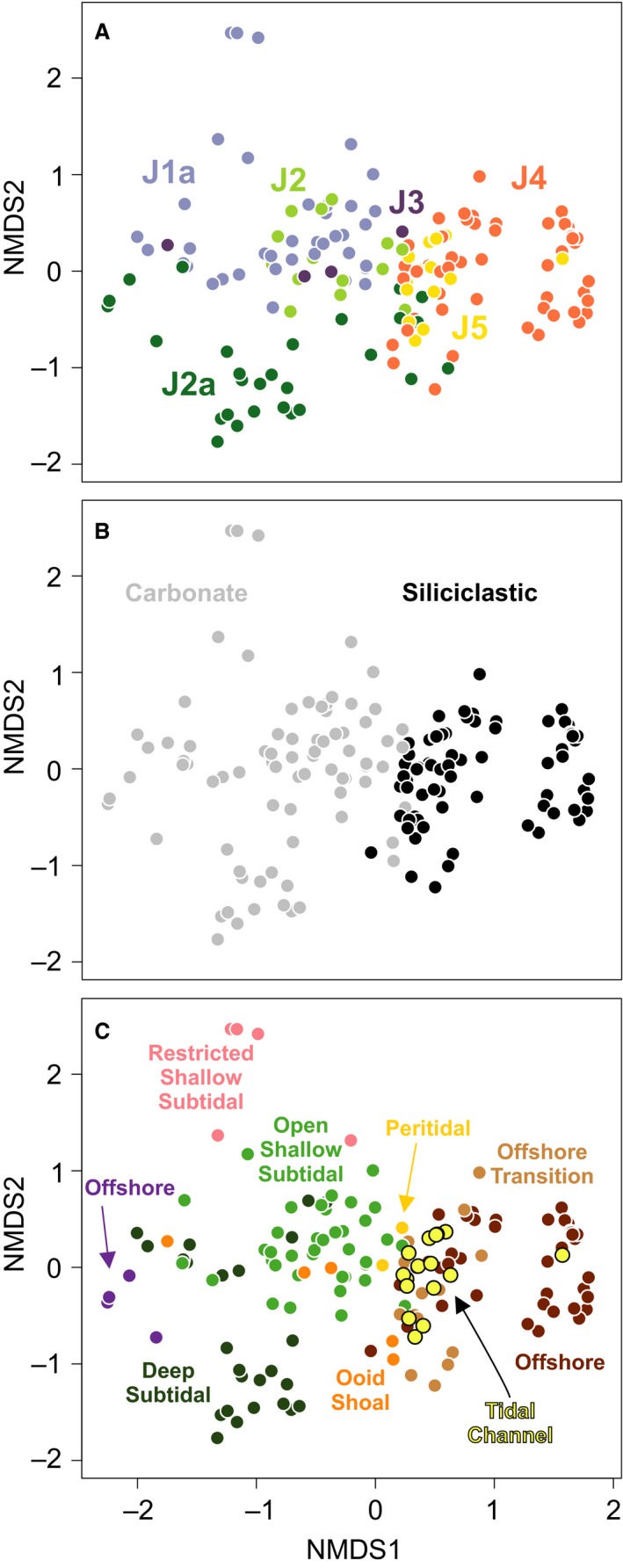
Nonmetric multidimensional scaling (NMDS) of fossil assemblages from the entire studied interval. A, sample scores coded by depositional sequence. B, sample scores coded by carbonate and siliciclastic depositional systems. C, sample scores coded by depositional environment.

The NMDS ordination followed by PCA rotation places samples within a space such that the relative positions of samples reflect their similarity and such that axis 1 of the ordination explains the greatest amount of variation, followed by axis 2, and so on. Coding the samples by depositional sequence, lithological system, and depositional environment is used to interpret the sources of variation underlying these axes (Fig. 4).

When coded by depositional sequence (Fig. 4A), Middle Jurassic samples have low to intermediate axis 1 scores, and Upper Jurassic samples have intermediate to high axis 1 scores. Coding by lithological system (Fig. 4B) reveals that samples with low axis 1 scores are from carbonate systems and those with high axis 1 scores are from siliciclastic systems, reflecting the long‐term transition within the Sundance Seaway (McMullen *et al*. 2014). In detail, sequences J1a and J2 are exclusively composed of carbonates, J2a contains both carbonates and siliciclastics, J3 only includes carbonates, and J4 and J5 are exclusively siliciclastic, except from three samples from a J4 ooid shoal.

Coding by depositional environment reveals a second pattern superimposed on this overall trend (Fig. 4C). Carbonate deep‐water samples (offshore and deep subtidal) have low axis 1 scores, while deep‐water siliciclastic samples (offshore) have the highest axis 1 scores. The shallowest environments of both carbonate and siliciclastic systems (open and restricted shallow subtidal, ooid shoal, peritidal, tidal channel, offshore transition) have intermediate axis 1 scores and plot in the middle of the ordination. Although this pattern indicates that water depth is expressed on axis 1, as is common in ordinations of marine invertebrates (Patzkowsky & Holland 2012), it deviates in an important way from the typical pattern in which deep‐water and shallow‐water samples lie at opposite ends of axis 1. The pattern in this data indicates that shallow‐water samples are similar in taxonomic composition, regardless of their age or whether they derive from a carbonate or siliciclastic system. Furthermore, the strongest compositional difference in the entire data set is between deep‐water samples from the older carbonate systems and the younger deep‐water siliciclastic systems.

Axis 2 of the ordination reflects the combination of temporal changes in taxonomic composition and environmental conditions (Fig. 4). Within carbonate systems, the older J1a have large axis 2 scores, whereas the younger J2a samples have low axis 2 scores. J2 and J3 samples have intermediate scores. Among J2a samples, those from the deep subtidal have smaller axis 2 scores than those from the offshore. Within the open shallow subtidal samples, samples from the J1a and J2 sequences overlap along axis 2, indicating little faunal turnover in the open shallow subtidal of these sequences. In siliciclastic systems, offshore and offshore transition samples of the J4 sequence and tidal channel samples of the J5 sequence have highly variable axis 2 scores at low to intermediate values. This pattern suggests that a combination of temporal changes and lateral variations within the environments preserved along the onshore–offshore depth gradient (*see* Holland & Patzkowsky 2004).

### Ordination and cluster analysis of the Middle Jurassic

Because age and depositional system are intertwined (Fig. 4A, B), the data set was divided into an older (J1a–J3) primarily carbonate‐system portion and a younger (J4–J5) primarily siliciclastic‐system portion to characterize the community composition of each.

**Figure 5:**
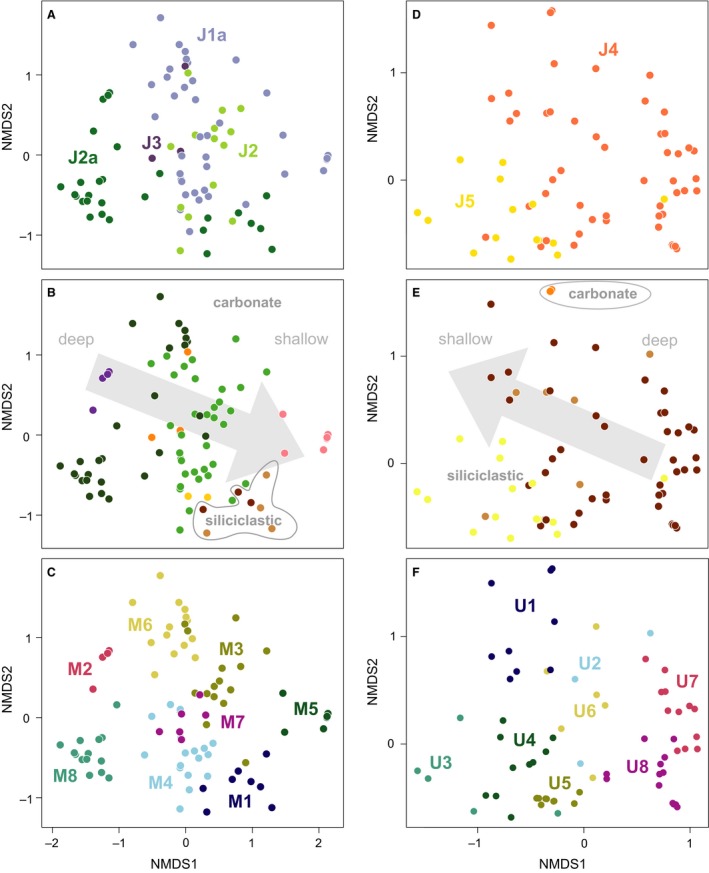
Nonmetric multidimensional scaling (NMDS) of fossil assemblages from Middle (J1a–J3) and Upper Jurassic (J4–J5) sequences, separately. A, D, sample scores coded by depositional sequence. B, E, sample scores coded by depositional environment, with colour codes as in Fig. 4C. C, F, sample scores coded by biofacies.

Ordination of the J1a–J3 samples (Fig. 5A, B) also reproduces a water depth gradient largely along axis 1, with offshore and deep subtidal samples at low axis 1 scores, and shallower samples (open shallow subtidal, restricted shallow subtidal, ooid shoal, peritidal) at higher axis 1 scores. Siliciclastic‐system J2a samples from offshore and offshore transition environments are the exception to this pattern, and they have high axis 1 scores, a pattern consistent with the ordination of all samples (Fig. 4). Samples also segregate by age, with the oldest samples (J1a) having relatively high scores on both axis 1 and axis 2, and J2a samples having relatively low scores on both axes. These differences again reflect turnover in the composition of deep subtidal communities. J2 samples tend to have high axis 1 scores, but low axis 2 scores, and the few J3 samples have intermediate scores. These differences in where samples of a given age plot mostly reflect differences in the environments preserved in each sequence (Fig. 3).

**Figure 6:**
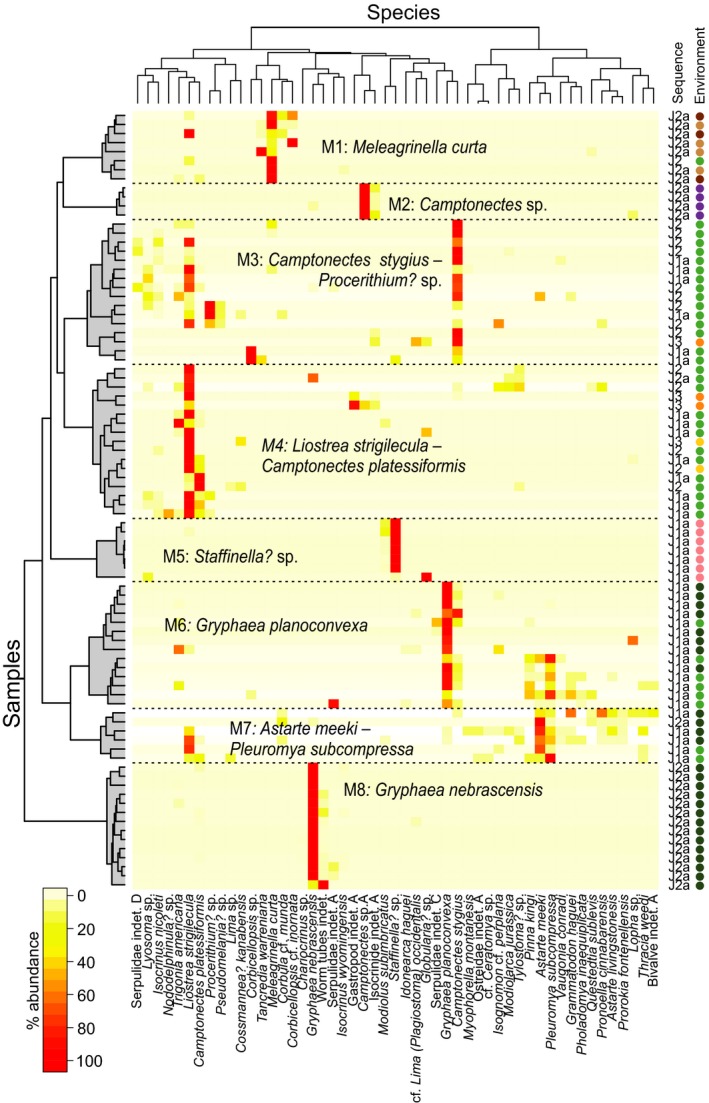
Heat‐map diagram of a two‐way hierarchical clustering analysis of samples from the Middle Jurassic (sequences J1a to J3). Environment colour codes as in Fig. 4.

Eight biofacies were identified with two‐way cluster analysis (Fig. 6, Table 1), and samples in the ordination were coded by biofacies (Fig. 5C) to show the relationships among these biofacies and how they relate to the main environmental gradient.

**Table 1:** Species composition of Middle Jurassic biofacies, with motility, tiering and feeding categories shown for each species

	%	Motility	Tiering	Feeding
Biofacies M1				
* Meleagrinella curta*	60.7	Stat	Epi	Susp
* Corbicellopsis* cf. *inornata*	12.4	Fac	Deep	Dep
* Liostrea strigilecula*	12.0	Stat	Epi	Susp
* Tancredia warrenana*	10.2	Fac	Deep	Dep
* Corbula* cf. *munda*	2.9	Stat	Shal	Susp
Biofacies M2				
* Camptonectes* sp.	90.1	Fac	Epi	Susp
* *Isocrinidae indet.	7.4	Stat	Epi	Susp
Biofacies M3				
* Camponectes stygius*	37.0	Fac	Epi	Susp
* Procerithium*? sp.	20.9	Slow	Epi	Graz
* Liostrea strigilecula*	19.6	Stat	Epi	Susp
* Lyosoma* sp.	3.1	Slow	Epi	Graz
* Pseudomelania*? sp.	2.9	Slow	Epi	?
* Corbicellopsis* sp.	2.8	Fac	Deep	Dep
* *Serpulidae indet. D	2.8	Stat	Epi	Susp
Biofacies M4				
* Liostrea strigilecula*	60.8	Stat	Epi	Susp
* Camponectes platessiformis*	12.3	Fac	Epi	Susp
* Trigonia americana*	5.5	Fac	Shal	Susp
* Procerithium*? sp.	5.4	Slow	Epi	Graz
Biofacies M5				
* Staffinella*? sp.	77.3	Fac	Shal	Susp
* Globularia*? sp.	17.1	Slow	Epi	Graz
* Modiolus subimbricatus*	2.7	Stat	Semi	Susp
* Lyosoma* sp.	2.7	Slow	Epi	Graz
Biofacies M6				
* Gryphaea planoconvexa*	59.4	Stat	Epi	Susp
* Camptonectes stygius*	8.0	Fac	Epi	Susp
* Pleuromya subcompressa*	7.6	Fac	Deep	Susp
* *Serpulidae indet. A	4.7	Stat	Epi	Susp
* Trigonia americana*	4.1	Fac	Shal	Susp
* Pinna kingi*	2.9	Stat	Semi	Susp
* *Serpulidae indet. C	2.3	Stat	Epi	Susp
* Lopha* sp.	2.1	Stat	Epi	Susp
Biofacies M7				
* Astarte meeki*	29.4	Fac	Shal	Susp
* Liostrea strigilecula*	14.2	Stat	Epi	Susp
* Pleuromya subcompressa*	13.7	Fac	Deep	Susp
* Grammatodon haguei*	4.7	Fac	Epi	Susp
* Pronoella cinnabarensis*	4.7	Fac	Shal	Susp
* Astarte livingstonensis*	4.3	Fac	Shal	Susp
* Corbula* cf. *munda*	4.3	Stat	Shal	Susp
* Thracia weedi*	3.8	Fac	Deep	Susp
Biofacies M8				
* Gryphaea nebrascensis*	95.5	Stat	Epi	Susp
* *Worm tubes indet.	2.3	Stat	Epi	Susp

Only species more abundant than 2% are shown. Motility: Stat, stationary; Fac, facultative mobile; Slow, mobile‐slow. Tiering: Epi, epifaunal; Shal, shallow‐infaunal; Semi, semi‐infaunal; Deep, deep‐infaunal. Feeding: Susp, suspension feeder; Dep, deposit feeder; Graz, grazer.

Most J1a–J3 biofacies are dominated by a single taxon (biofacies M1, M2, M4, M5, M6 and M8: Table 1, Fig. 6), and have low richness (<5) and evenness (<0.4) (Table 2). Only biofacies M3 and M7 are not so dominated by a single species (Table 1, Fig. 6); they have richnesses of 5 and 12 and Simpson's indices of 0.5 and 0.8, greater than the other six biofacies from J1a–J3.

**Table 2:** Biofacies richness and evenness

Biofacies	S	S_rar30_	S_pool_	S_SQS0.50_	1−D
U1	3.4	6.2	12	5.3	0.3
U2	6.3	9.2	15	4.0	0.6
U3	3.0	4.7	7	2.8	0.4
U4	2.5	3.8	9	4.9	0.2
U5	2.3	2.2	5	6.9	0.2
U6	6.5	10	19	9.9	0.7
U7	3.9	4.2	11	8.8	0.4
U8	3.2	4.1	7	5.5	0.3
M1	3.1	5.3	10	5.6	0.3
M2	2.5	2.7	4	2.1	0.2
M3	5.4	9.3	37	12.4	0.5
M4	4.1	8.2	28	14.9	0.4
M5	2.3	3.2	5	3.0	0.1
M6	5.5	8.8	29	14.0	0.5
M7	12.7	14.5	39	22.3	0.8
M8	3.3	2.1	11	5.5	0.1

*S*, mean sample richness; *S*_rar30_, sample richness, rarefied to 30 individuals per sample (as plotted in Fig. 8); *S*_pool_, total number of taxa in each biofacies; S_SQS0.5_, richness standardized by shareholder quorum subsampling with a quota of 0.5; 1‐D, mean sample evenness measured as the Simpson index of diversity (as plotted in Fig. 8).

Several J1a–J3 biofacies are limited to particular combinations of depositional sequence and environment (Fig. 5B, C). For example, biofacies M2 is limited to carbonate offshore facies of J2a; M5 is found only in the restricted shallow subtidal of J1a; and M8 is present exclusively in the deep subtidal of sequence J2a. Biofacies M6 occurs only in the deep subtidal and shallow subtidal of J1a, and the diverse bivalve biofacies M7 is almost as restricted but is also found in one deep subtidal sample from the J2a.

Three of the biofacies are more widely distributed (Fig. 5B, C). Biofacies M1 occurs in the J2 siliciclastic system and was also found in one open shallow subtidal sample from the J1a. The diverse biofacies M3 occurs frequently in the open shallow subtidal samples of J1a and J2, and more rarely in the ooid shoal of J3. Dominated by the small oyster *Liostrea strigilecula*, biofacies M4 is present mostly in the open shallow subtidal zone of J1a and J2, but also in the open shallow subtidal environment of J2a, the peritidal of J2 and J3, and the ooid shoal of J3.

Biofacies are arrayed with overlap along the depth gradient (Fig. 5C). Biofacies M2, M6, and M8 have relatively low axis 1 scores and therefore are interpreted as relatively deeper‐water biofacies. Biofacies M3, M4, and M7 occupy intermediate positions along the depth gradient, and biofacies M1 and M5 have the highest axis 1 scores and are interpreted as relatively shallow‐water biofacies. Most biofacies overlap in ordination space, except for M2, M5, and M8, consistent with these biofacies representing somewhat artificial divisions of a biotic gradient, rather than discrete community types, an interpretation consistent with the substantial taxonomic overlap in the composition of these biofacies (Table 1, Fig. 6).

### Ordination and cluster analysis of the Upper Jurassic

A water depth gradient is also recognizable in the ordination of J4 and J5 sequences, again along axis 1, indicating that water depth is the primary source of variation in community composition (Fig. 5E). Tidal channel samples have the lowest axis 1 scores, offshore transition samples have intermediate axis 1 scores, and offshore samples have the highest axis 1 scores. The only two carbonate samples, both from J4, plot at intermediate axis 1 scores but the highest axis 2 scores.

**Figure 7:**
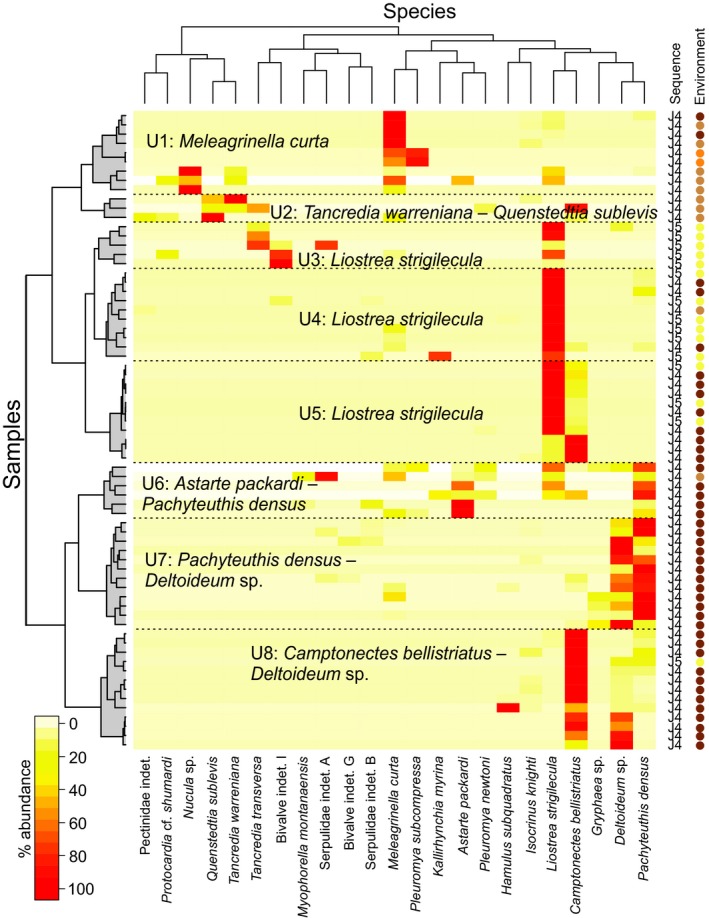
Heat‐map diagram of a two‐way hierarchical clustering analysis of samples from the Upper Jurassic (sequences J4–J5). Environment colour codes as in Fig. 4.

Eight biofacies are identified in the two‐way cluster analysis (Fig. 7; Table 3) and plotting these on the ordination shows their interrelationships (Fig. 5F).

**Table 3:** Species composition of Upper Jurassic biofacies, with motility, tiering and feeding strategies as in Table 1

	%	Motility	Tiering	Feeding
Biofacies U1				
* Meleagrinella curta*	60.9	Stat	Epi	Susp
* Nucula* sp.	20.7	Fac	Shal	Dep
* Liostrea strigilecula*	7.3	Stat	Epi	Susp
* Pleuromya subcompressa*	6.3	Fac	Deep	Susp
* Tancredia warrenana*	2.2	Fac	Deep	Dep
Biofacies U2				
* Tancredia warrenana*	37.1	Fac	Deep	Dep
* Quenstedtia sublevis*	32.3	Fac	Shal	Dep
* Camptonectes bellistriatus*	12.9	Fac	Epi	Susp
* Tancredia transversa*	6.5	Fac	Deep	Dep
* Meleagrinella curta*	3.2	Stat	Epi	Susp
* *Pectinidae indet.	3.2	Fac	Epi	Susp
Biofacies U3				
* Liostrea strigilecula*	41.7	Stat	Epi	Susp
* *Bivalve indet. I	34.6	?	?	?
* Tancredia transversa*	16.1	Fac	Deep	Dep
* *Serpulidae indet. A	3.3	Stat	Epi	Susp
* Deltoideum* sp.	2.4	Stat	Epi	Susp
Biofacies U4				
* Liostrea strigilecula*	83.5	Stat	Epi	Susp
* Kallirhynchia myrina*	9.1	Stat	Epi	Susp
Biofacies U5				
* Liostrea strigilecula*	63.6	Stat	Epi	Susp
* Camptonectes bellistriatus*	35.9	Fac	Epi	Susp
Biofacies U6				
* Astarte packardi*	31.4	Fac	Shal	Susp
* Pachyteuthis densus*	20.8	Fast	Nekt	Carn
* Liostrea strigilecula*	11.8	Stat	Epi	Susp
* *Serpulidae indet. A	9.4	Stat	Epi	Susp
* Meleagrinella curta*	6.5	Stat	Epi	Susp
* Camptonectes bellistriatus*	3.7	Fac	Epi	Susp
* Pleuromya newtoni*	3.7	Fac	Deep	Susp
* Myophorella montanaensis*	3.3	Fac	Shal	Susp
* *Serpulidae indet. A	2.9	Stat	Epi	Susp
* Kallirhynchia myrina*	2.4	Stat	Epi	Susp
* Pleuromya subcompressa*	2.0	Fac	Deep	Susp
Biofacies U7				
* Pachyteuthis densus*	61.1	Fast	Nekt	Carn
* Deltoideum* sp.	30.8	Stat	Epi	Susp
* Gryphaea* sp.	2.4	Stat	Epi	Susp
Biofacies U8				
* Camptonectes bellistriatus*	76.6	Fac	Epi	Susp
* Deltoideum* sp.	14.3	Stat	Epi	Susp
* Hamulus subquadratus*	4.7	Stat	Epi	Susp
* Pachyteuthis densus*	2.7	Fast	Nekt	Carn

Only species more abundant than 2% are shown. Motility: Stat, stationary; Fac, facultative mobile; Fast, mobile‐fast. Tiering: Epi, epifaunal; Shal, shallow‐infaunal; Deep, deep‐infaunal; Nekt, nekto‐benthic. Feeding: Susp, suspension feeder; Dep, deposit feeder; Carn, carnivore.

Five of these eight biofacies are dominated by a single taxon, as seen in the Middle Jurassic sequences (biofacies U1, U4, U5, U7, U8: Table 3, Fig. 7). These biofacies also have low richness (<4), and evenness (<0.4; Table 2). The other three biofacies (biofacies U2, U3, U6) have greater evenness (>0.4) and generally greater richness (two of the three have richness >6).

Many of the biofacies in J4–J5 are also restricted to particular depositional environments or sequences. For example, biofacies U2 is limited to the J4 offshore transition, U3 to J5 tidal channels, and U6 and U7 to the J4 offshore. Biofacies U8 has almost the same distribution as U6 and U7, but was also found in one J5 tidal channel sample. The remaining three biofacies are more broadly distributed. Biofacies U1 occurs mostly in the J4 offshore transition and ooid shoal, but also rarely in the offshore. Biofacies U4 occurs in tidal channel, offshore and offshore transition facies of sequence J4 and J5. Biofacies U5 occurs in the J4 offshore and in J5 tidal channels.

As for the Middle Jurassic, Upper Jurassic biofacies are also distributed along a depth gradient, with overlap of many of the biofacies in ordination space (Fig. 5F). Biofacies U7 and U8 have the highest axis 1 scores and are interpreted as deeper‐water biofacies, whereas U1 and U3 have the lowest axis 1 scores and are interpreted to be shallower‐water biofacies. All remaining biofacies (U2, U4, U5, U6) occupy intermediate positions. With a few exceptions (e.g. U3), most biofacies overlap in ordination space to some extent, indicating the gradient nature of community composition.

### Changes in richness and evenness

**Figure 8:**
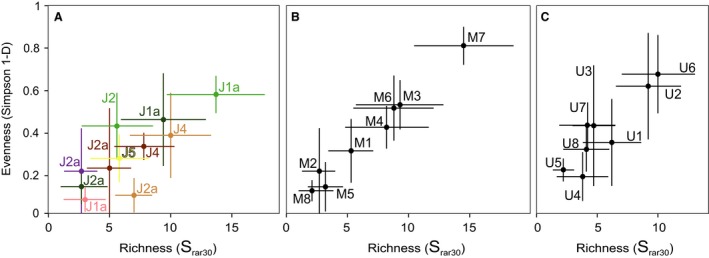
Sample richness, rarefied to 30 individuals per sample (*S*_rar30_) and evenness (Simpson 1‐D) of samples by depositional environment and biofacies, with 95% confidence intervals. A, samples grouped by depositional environment, showing only those that have more than three samples and with colour codes as in Fig. 4C. B, Middle Jurassic samples grouped by biofacies. C, Upper Jurassic samples grouped by biofacies.

When the data are binned by depositional sequence and environment (Fig. 8A), the highest rarefied richness and evenness occurs in the deep subtidal and open shallow subtidal of sequence J1a and in the offshore and offshore transition of sequence J4. The open shallow subtidal of J2 also has high evenness but intermediate richness. In contrast, sequence J2a contains many of the lowest values of richness and evenness, both in samples from carbonate (deep subtidal and offshore) and siliciclastic (offshore and offshore transition) systems. These low values are comparable to those of the restricted shallow subtidal, which would be expected to have low diversity because of its hypersalinity (Clement & Holland 2016).

Richness and evenness are similar in most Middle and Upper Jurassic biofacies, except for the unusually rich and even M7 biofacies that contains a diverse suite of bivalves (Fig. 8B, C, Tables 1, 2).

## Discussion

### Diversity and evenness in the Sundance Seaway

The southern Sundance Seaway records benthic communities from a wide array of depositional environments, ranging from restricted coastal settings to fully open‐marine conditions. Diversity and evenness patterns are controlled in large part by depositional environment, with open‐marine communities showing higher richness and evenness than communities from restricted or marginal environments, such as the restricted shallow subtidal or tidal channel (Fig. 8). In addition, richness and evenness tend to be higher in carbonate systems than in siliciclastic systems. One exception to these patterns is sequence J2a, which has low richness and evenness even in open marine facies, and in both carbonate and siliciclastic systems.

This low evenness in the Sundance Seaway is reflected in the structure of biofacies, which are often dominated by one or two species that constitute 60–95% of the community. Some of these species (e.g. *Liostrea strigilecula* and *Meleagrinella curta*) are found in multiple biofacies, depositional environments, and sequences, and they persisted from the initiation to the final filling of the Seaway. Other species are closely associated with a single depositional environment or sequence, such as *Staffinella*? sp. in the restricted shallow subtidal of J1a (biofacies M5) and *Gryphaea nebrascensis* in the deep shallow subtidal of J2a (biofacies M8). The few relatively diverse biofacies typically span adjacent depositional environments along an environmental gradient.

Low diversity, high dominance assemblages of body fossils and trace fossils have been recognized previously in the Sundance Seaway (Wright 1973, 1974; de Gibert & Ekdale 1999; McMullen *et al*. 2014) and this has been interpreted as a reflection of the restriction of the Seaway and the resulting stresses imposed by wide variations in salinity and temperature (Peterson 1994; Tang & Bottjer 1996; Stanley 2010; McMullen *et al*. 2014). For instance, hypersaline conditions in areas characterized by coastal sabkhas and evaporative tidal flats could have affected the fauna living in adjacent subtidal areas (de Gibert & Ekdale 1999). Other factors could also have contributed to the low richness and evenness of the Sundance fauna. In particular, the peculiar palaeogeography of the Sundance Seaway, with its single entrance located at high latitude, 2000 km from its southern terminus, coupled with its shallow depth, could have hindered the immigration of species from the open ocean, thus reducing the diversity of body and trace fossils (Tang & Bottjer 1996; de Gibert & Ekdale 1999).

The richness of the Sundance fauna, even for biofacies deposited under normal marine conditions, is low compared with other regional studies on Middle–Upper Jurassic benthic communities (e.g. Fürsich 1977, 1984; Fürsich & Heinberg 1983; Oschmann 1988; Wignall 1990; Abdelhady & Fürsich 2014). In his study on the biogeography of Jurassic bivalves, Hallam (1977) identified the Middle Jurassic West American Province, also called Shoshonean Province (Taylor *et al*. 1984), as an impoverished version of the European Province. Similarly, within‐biofacies diversity is generally lower (*S*_pool_, Table 2) than the median richness of 21.5 from comparable settings globally (Bambach 1977). The only Sundance biofacies that exceed this (M3, M4, M6, M7) all come from the Middle Jurassic carbonate system, in particular the deep subtidal and open shallow subtidal environments, suggesting that at least some Sundance settings favoured high diversity. The rarefied richness of most Sundance biofacies is also less than the Mesozoic average rarefied richness of 7.12 (Kowalewski *et al*. 2006). The same four Middle Jurassic biofacies (M3, M4, M6, M7) exceed this average, together with one Upper Jurassic one (U2, Table 2).

### Environmental controls on benthic community changes through time

Throughout the Jurassic in the Sundance Seaway, benthic communities varied in composition along a water‐depth gradient that reflects onshore–offshore position, and this pattern is shown by the distribution of biofacies (Fig. 5). In the Middle Jurassic, biofacies M2 (*Camptonectes* sp.), M6 (*Gryphaea planoconvexa*), and M8 (*G*. *nebrascensis*) occur mostly in deeper water environments (carbonate deep subtidal and offshore), with the biofacies M2 also occurring in the open shallow subtidal of sequence J1a. Biofacies M3 (*Camptonectes stygius* and *Procerithium?* sp.), M4 (*Liostrea strigilecula* and *Camptonectes platessiformis*) and M7 (*Astarte meeki* and *Pleuromya subcompressa*) occur mostly in open shallow subtidal facies, but also in the adjoining ooid shoal and peritidal environments. Biofacies M3 and M4 also record the highest abundance and diversity of epifaunal grazing gastropods, possibly indicating a position well within the photic zone, where algal growth increased the complexity of the habitat (Fürsich 1984). Restricted shallow subtidal facies, which formed under elevated salinity conditions in the most inner part of the carbonate ramp during sequence J1a (Clement & Holland 2016), lie at the shallow extreme of the gradient. Biofacies M1 is most common in samples from siliciclastic systems, but occur also in open shallow subtidal facies of sequences J2, highlighting the eurytopic character of its dominant species *M. curta*.

In the Upper Jurassic, the shallowest biofacies along the onshore–offshore gradient are the *M. curta* (U1), *T. warrenana – Q. sublevis* (U2), and *Liostrea strigilecula* (U3) biofacies. The U3 biofacies occurs only in tidal facies assemblages of sequence J5, whereas U1 and U2 occur in offshore transition and ooid shoal facies of J4, and rarely in the offshore. Two other biofacies rich in *Liostrea strigilecula* (U4 and U5) are shared among tidal channel, offshore transition and offshore facies, whereas biofacies U6, U7, and U8 only occur in the offshore of J4. This trend is consistent with the interpretation of *Liostrea* as a eurytopic genus, able to tolerate a wide range of habitats, especially changes in salinity (Hallam 1977).

Water depth is interpreted as one of the most important factors describing the distribution of marine benthic organisms, as shown in multiple studies from modern and ancient settings (e.g. Scarponi & Kowalewski 2004; Holland & Patzkowsky 2004; Hohenegger 2005; Dominici *et al*. 2008; Danise *et al*. 2013; reviewed in Patzkowsky & Holland 2012). This pattern is caused by the correlation with water depth of the many physical factors that control the structure and taxonomic composition of level‐bottom communities, such as temperature, salinity, water energy, substrate consistency, and grain size (Patzkowsky & Holland 2012). Such a relationship between assemblages and water depth can only be stable over geological time if the relationships of environmental factors with water depth remain consistent over broad geographical ranges and long spans of time. At larger spatial and longer temporal scales, other factors can control faunal distributions, such as provincialism (Patzkowsky & Holland 2012). In the Sundance Seaway, the onshore–offshore gradient is preserved through four third‐order depositional sequences in the Middle Jurassic, (J1a, J2, J2a, J3), which span an interval of time of ~5 myr. In the Upper Jurassic, a relatively consistent gradient is present through two third‐order depositional sequences (J4 and J5) that span the entire Oxfordian (~6 myr).

Over the entire marine history of the Sundance Seaway (~13 myr), the main environmental factor controlling the distribution of faunal community is the change in depositional system, from carbonate to siliciclastic. In the ordination of the complete data set (Fig. 4), Middle Jurassic samples (J1a–J3) plot separately from Upper Jurassic ones (J4–J5) along axis 1, showing that the main ecological transition occurred around the Callovian to Oxfordian boundary. A previous ordination of a much smaller Sundance data set (McMullen *et al*. 2014) also identified this transition as the primary factor controlling the distribution of taxa through time. In our analysis, the only exception to this temporal trend is the presence of siliciclastic offshore and offshore transition samples in the Bathonian sequence J2a, which have a similar taxonomic composition to offshore transition samples of sequence J4 and plot with Upper Jurassic samples (Fig. 4). These samples all belong to a *Meleagrinella curta* biofacies (M1 and U1; Figs 5, 6, 7), which persisted across the J4 unconformity, probably the longest hiatus in the history of the Seaway (at least 2 myr: Imlay 1980). The importance of the J4 transition is also reflected in the lithostratigraphical nomenclature of the Sundance Seaway throughout the region. In Wyoming, the Sundance is often informally divided into an upper and lower Sundance across this boundary (Kvale *et al*. 2001). In Montana, this boundary separates the underlying carbonate‐dominated Rierdon Formation from the overlying siliciclastic‐dominated Swift Formation (Imlay 1962; Parcell & Williams 2005). In Idaho, this boundary separates the carbonate Twin Creek Formation from the Preuss and Stump formations (Imlay 1967).

This switch in depositional system was likely to have been driven by the gradual northward migration of the North American plate during the Jurassic, which moved the study area out of the subtropical latitudes that fostered an arid climate and carbonate–evaporite deposition, and into one with progressively more humid climates (Johnson 1992; Rees *et al*. 2000; Boucot *et al*. 2013) with increased weathering and siliciclastic deposition. By the end of the Jurassic, the region also moved from the belt of easterly tropical trade winds to the belt of the cooler westerlies in the middle latitudes (Kocurek & Dott 1983). This shift in depositional system and the accompanying faunal change may have also been enhanced by climate cooling, recorded at the Callovian–Oxfordian boundary interval in the northern hemisphere. Oxygen‐isotope data from English and Russian belemnites indicate a drop in temperature commencing in the latest Callovian (Jenkyns *et al*. 2002), and shark teeth from England, France and Switzerland indicate at 7°C drop in temperature at this time (Lécuyer *et al*. 2003). Coincident with this temperature decline, Boreal ammonite species invaded into lower latitude zones. Because regional facies analysis indicates sea‐level fall across the stage boundary, it has been suggested that this interval records build‐up of continental polar ice (Dromart *et al*. 2003).

As in our case‐study, another regional‐level palaeoecological analysis also found that a shift from a carbonate‐dominated to a siliciclastic‐dominated system was the main controlling factor in the ordination of samples (Bonelli & Patzkowsky 2008). On larger spatial and temporal scales substrate affinity of benthic organisms, and the availability of carbonate versus siliciclastic lithology through time, is considered to be a main driver in diversity and turnover dynamics, both in the Palaeozoic and Mesozoic (Miller & Connolly 2001, Kiessling & Aberhan 2007, Peters 2008). In particular, a study on the environmental affinity of Palaeozoic genera has shown that differences in dynamic between carbonate and siliciclastic lovers act especially on relatively short time scales (<5 myr), and could be driven by brief environmental perturbations that preferentially affect carbonate systems (Foote 2006). Carbonate systems are volatile, being sensitive to changes in temperature, nutrient input, as well as siliciclastic supply. Siliciclastics in particular can inhibit or dilute carbonate production. Increases in terrigenous sediment supply, which can be driven by sea‐level fall, tectonic uplift, and climate change, can result in the selective elimination of carbonate environments relative to siliciclastics, and drive ecological and evolutionary change (Peters 2008).

### Differential onshore–offshore faunal turnover

**Figure 9:**
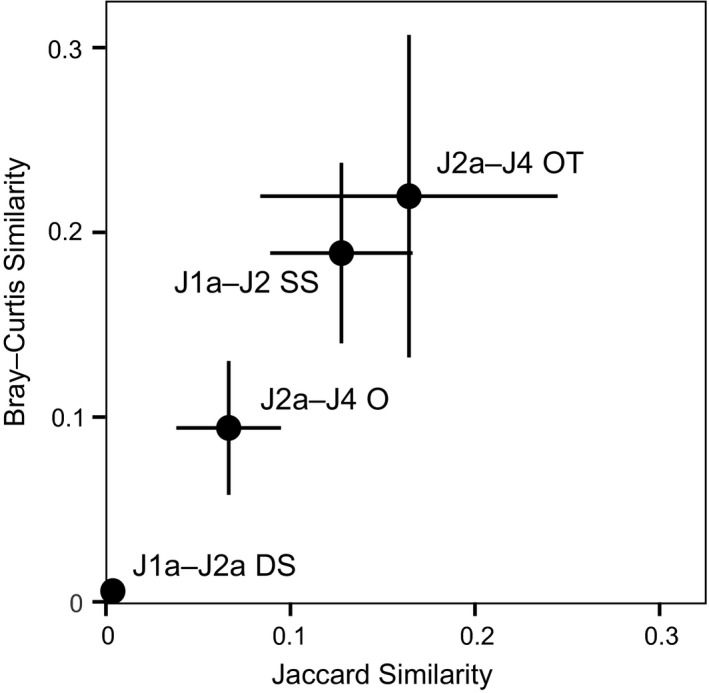
Mean Jaccard and Bray–Curtis similarities as measures of taxonomic turnover within equivalent depositional environments of different depositional sequences, with 95% confidence intervals. A similarity index of 0 corresponds to samples sharing no taxa, and an index of 1 reflecting identical samples. *Abbreviations*: DS, deep subtidal; SS, open shallow subtidal; O, offshore; OT, offshore transition.

Ordination of faunal censuses from the Sundance Seaway demonstrate the sustained similarity of shallow‐water biofacies and the greater turnover in deeper‐water biofacies. This onshore–offshore variation in turnover is observed both between successive third‐order depositional sequences within the same depositional system (carbonate or siliciclastic), and in the transition from the carbonate system to the siliciclastic system. Both the Jaccard and the Bray–Curtis similarity measures indicate that taxonomic similarity decreased from onshore to offshore in successive third‐order depositional sequences, although similarity values are low for both onshore and offshore environments (Fig. 9). Turnover is higher offshore in carbonate (open shallow subtidal versus deep subtidal environments) as well as siliciclastic systems (offshore vs offshore transition environments), with J1a and J2a deep subtidal environments having the lowest levels of mean similarity (Fig. 9). These patterns suggest that onshore communities were more resilient to perturbations caused by third‐order sea‐level changes compared with offshore communities. At the same time, onshore communities were also more resilient to the switch from carbonate to siliciclastic deposition at the Middle–Upper Jurassic transition. This is shown by the ordination of all samples, where onshore samples from carbonate and siliciclastic systems plot one close to the other, indicating higher taxonomic similarity compared to offshore ones, which plot at the opposite ends of NMDS axis 1 (Fig. 4).

The higher resilience of onshore biofacies to third‐order sea‐level fluctuations and to the change from carbonate to siliciclastic systems was driven by a few, dominant, eurytopic species. Abundant species like *Liostrea strigilecula* and *Meleagrinella curta* were eurytopic and not restricted to a single facies, sequence or depositional environment, and they were long‐lived, persisting from the opening to the closing of the Seaway (from J1a to J5). Turnover, which mostly occurred at the species level, occurred mainly in taxa adapted to a more restricted range of habitats, and these species were more abundant in more seaward settings. For instance, the species *Gryphaea planoconvexa*, common in deep and shallow subtidal environments of J1a (biofacies M6), was replaced by *G. nebrascensis* in the deep subtidal of J2a (biofacies M8), and by *Gryphaea* sp. in the offshore of J4 (biofacies U7). The pectinid *Camptonectes* is present in open shallow subtidal, ooid shoal and peritidal environments of J1a to J2a as *C. platessiformis* (biofacies M4), in deep and shallow subtidal environments of J1a to J3 as *C. stygius* (biofacies M3–M6), in offshore carbonate facies of J1a as *Camptonectes* sp. (biofacies M2), and in offshore siliciclastic facies of J4 as *Camptonectes bellistriatus*.

Previous studies have shown that shallow‐water settings are numerically dominated by abundant, eurytopic, geographically widespread, and geologically long‐lived species, whereas offshore faunas tend to be dominated by species that are less abundant, more stenotopic, geographically restricted, and geologically short‐lived (e.g. Jackson 1974, Jablonski & Valentine 1981, Kammer *et al*. 1997). Somewhat paradoxically, taxonomic extinction (Sepkoski 1987) and origination (Kiessling & Aberhan 2007) rates increase onshore. These apparently conflicting observations are reconciled by a model in which preferential onshore extinction leads to the accumulation of extinction‐resistant clades (i.e. eurytopic, geographically widespread) that spread offshore over macroevolutionary timescales (Sepkoski 1981). The cause of enhanced onshore extinction has been attributed to greater short‐term environmental variability (Sepkoski 1987) and to greater onshore variability in habitat area during relative sea‐level changes (Holland & Christie 2013). Biofacies composition and change in the Sundance Seaway follows these same patterns, with onshore faunas characterized by abundant and long‐lived species relative to those offshore. Thus, in the Sundance biofacies show relatively greater turnover in the offshore than the onshore. These results emphasize the contrast between taxon‐based approaches, which indicate greater extinction and turnover onshore, and ecological approaches based on relative abundance, which show greater turnover offshore. These results match well previous studies that have shown how ecologically‐based analyses of macroevolutionary patterns differ from taxonomically‐based analyses (Boucot 1983; McGhee *et al*. 2004; Christie *et al*. 2013; Dineen *et al*. 2014). Tomašových *et al*. (2014) found no consistent onshore–offshore patterns of turnover within Eocene and Pliocene stages, but elevated onshore turnover from the Eocene to the Pliocene that was driven by selectivity in onshore regions, underscoring that global macroevolutionary patterns are driven by regional‐scale patterns of selectivity whose effects accumulate over long spans of time.

Our results partly conflict with a previous interpretation of faunal stasis in the Jurassic Sundance Seaway (Tang & Bottjer 1996). The authors, using mostly presence–absence data, and without differentiating between depositional environments, found that many bivalve species and associations persisted through sea‐level and environmental changes, through depositional units and stratigraphical breaks, with no turnover events. They attributed this stability to the environmental conditions of the Jurassic Sundance Seaway that selected for generalist taxa capable of withstanding environmental disturbances and persisting for long intervals of time (Tang & Bottjer 1996). Our analysis also finds that many species are persistent through the history of the Sundance Seaway, but mainly in onshore settings. Faunas of offshore settings change not only with the switch from a carbonate system to a siliciclastic one, but also from one depositional sequence to the next. Recognizing this turnover requires the use of quantitative abundance data, comparisons within sedimentologically defined depositional environments, and a sequence stratigraphic framework.

## Conclusions

The use of quantitative abundance data integrated within a time–environment framework reveals the change of benthic communities of the Middle–Upper Jurassic Sundance Seaway from its opening to its closure, over a time interval of about 13 myr. The Sundance Seaway is characterized by communities with low richness and high dominance, unlike most Jurassic communities worldwide. This diversity structure may have resulted from the unusual physiography of the seaway, whose sole connection to the Pacific Ocean was about 2000 km north of its southern end. This probably fostered strong gradients along the axis of the seaway in temperature and salinity that hindered the immigration of species from the open ocean.

Communities within the Sundance Seaway were arrayed along a water depth gradient that changed over the history of the seaway. In the Middle Jurassic (Bajocian–Callovian sequences J1a–J3) benthic communities occupied a carbonate ramp, but in the Upper Jurassic (Oxfordian sequences J4 and J5), communities were developed along a siliciclastic shelf. This transition to a siliciclastic system triggered turnover in the benthic fauna, with greater turnover in offshore environments relative to onshore environments. Higher turnover in more seaward areas is present not only at the Middle to Upper Jurassic transition, but also among successive depositional sequences in general. The higher resilience of onshore communities to sea‐level variations and to the switch from a carbonate system to a siliciclastic one was controlled by a few abundant eurytopic species (e.g. *Ostrea strigilecula* and *Meleagrinella curta*). Lower stability in offshore settings was controlled by the greater stenotopy of species (e.g. species of the genus *Gryphaea* or *Camptonectes*), which underwent higher turnover through time.

These results, based on an ecological analysis of relative abundance patterns, contrast with taxonomic‐based approaches and indicate the need for ecological studies to complement taxonomic studies of macroevolutionary events. This study shows how a stratigraphic palaeobiological approach is essential for understanding the link between environmental and faunal gradients, and for understanding the long‐term changes in these gradients over time that produce the local stratigraphical pattern of changes in community composition.
